# Optogenetic activation of dopamine D1 receptors in island cells of medial entorhinal cortex inhibits temporal association learning

**DOI:** 10.1186/s13041-023-01065-3

**Published:** 2023-11-14

**Authors:** Jun Yokose, Naoki Yamamoto, Sachie K. Ogawa, Takashi Kitamura

**Affiliations:** 1 Department of Psychiatry, University of Texas Southwestern Medical Center, Dallas, TX 75390 USA; 2 Department of Neuroscience, University of Texas Southwestern Medical Center, Dallas, TX 75390 USA

**Keywords:** Medial entorhinal cortex, Hippocampus, Trace fear conditioning, Optogenetics, Island cells, Dopamine D1 receptor, c-Fos

## Abstract

A critical feature of episodic memory formation is to associate temporally segregated events as an episode, called temporal association learning. The medial entorhinal cortical-hippocampal (EC-HPC) networks is essential for temporal association learning. We have previously demonstrated that pyramidal cells in the medial EC (MEC) layer III project to the hippocampal CA1 pyramidal cells and are necessary for trace fear conditioning (TFC), which is an associative learning between tone and aversive shock with the temporal gap. On the other hand, Island cells in MECII, project to GABAergic neurons in hippocampal CA1, suppress the MECIII input into the CA1 pyramidal cells through the feed-forward inhibition, and inhibit TFC. However, it remains unknown about how Island cells activity is regulated during TFC. In this study, we report that dopamine D1 receptor is preferentially expressed in Island cells in the MEC. Optogenetic activation of dopamine D1 receptors in Island cells facilitate the Island cell activity and inhibited hippocampal CA1 pyramidal cell activity during TFC. The optogenetic activation caused the impairment of TFC memory recall without affecting contextual fear memory recall. These results suggest that dopamine D1 receptor in Island cells have a crucial role for the regulation of temporal association learning.

**Supplementary Information:**

The online version contains supplementary material available at 10.1186/s13041-023-01065-3.

## Main

Remembering timing of distinct events and associating temporally discontinuous events are crucial process for the formation of episodic memories in daily life. We refer to this aspect of memory encoding as temporal association learning [1]. Pavlovian trace fear conditioning (TFC) have been established as one suitable animal models for temporal association learning [2]. The entorhinal cortical-hippocampal (EC-HPC) networks is currently considered to bridge and regulate the temporal discontinuity [3]. We have previously demonstrated that pyramidal cells in the medial EC (MEC) layer III project to the dorsal hippocampal CA1 (dCA1) pyramidal cells and are necessary for TFC [4]. On the other hand, pyramidal cells in MECII, which express both CalbindinD-28 K (CalB) and Wolfram syndrome 1 (Wfs1) (we refer to these as Island cells) [4, 5], project to GABAergic neurons in hippocampal dCA1, suppress the MECIII input into the dCA1 pyramidal cells through the feed-forward inhibition, and regulate TFC [4, 6, 7]. These results suggest a disinhibition model to control TFC, driving TFC by MECIII inputs into dCA1 and regulating TFC by Island cell inputs into dCA1. However, it remains unknown about how Island cell activity is regulated for successful association during TFC.

Dopaminergic projection in the brain play pivotal roles in various brain functions including temporal association learning, working memory, motivation, and motor control [8]. Dopaminergic projections into the prefrontal cortex, amygdala and basal ganglia have significant influence on working memory and fear conditioning [9]. In temporal association learning, the dopamine transporter hetero knockout mice showed the deficit in TFC [10]. While the dopaminergic innervation of the EC has been well characterized in rodents and monkeys [11, 12], the contributions of the dopamine receptors in MEC on TFC remain unexplored.

**Fig. 1:**
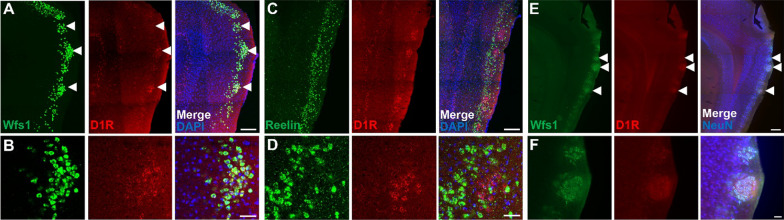
Island cells preferentially express dopamine D1 receptor in MEC. **A** Parasagittal sections of MEC fluorescence double in situ hybridization with anti-Wfs1 (green) and anti–D1R (red). **B** Magnified image from A. Arrowheads indicates Wfs1^+^/D1R^+^ cell-clusters. **C** Parasagittal sections of MEC fluorescence double in situ hybridization with anti-Reelin (green) and anti–D1R (red). **D** Magnified image from C. Fluorescence double in situ hybridization for D1R, Wfs1 and Reelin mRNA. MECII Wfs1^+^ cells, but not Reelin^+^ cells, express D1R. **E** Parasagittal sections of MEC immunostained with anti-Wfs1 (green) and anti–D1R (red). **F** Magnified image from **E**. Arrowheads indicates Wfs1^+^/D1R^+^ cell-clusters. Scale bar, 200 μm in **A**, **C** and **E**, 50 μm in **B**, **D** and **F**.

In this study, we first investigated the expression of dopamine D1 receptor in the MEC of 6–10 weeks old C57BL/6 J male and female mice (JAX: 000664). By using a specific in situ hybridization (ISH) probe for mRNA of dopamine D1 receptors (D1R) [13], we identified that D1R mRNA was localized in the layer II of MEC and made Island-like clusters similar to the distribution of Wfs1^+^ cells in MEC (Fig. 1A). Fluorescent double ISH revealed that Wfs1^+^ cells express D1R (Fig. 1B), while we could only detect a little D1R expression in Reelin^+^ cells (Fig. 1C, D) (the percentage of D1R + /Wfs1 + and D1R + /Reelin + cells were 89.5 ± 0.027%, total 617 cells from 4 slices and 3.95 ± 0.002%, total 1065 cells from 3 slices, respectively). ISH probes for Wfs1 and Reelin were labelled with fluorescein isothiocyanate (FITC). The ISH probe for D1R [13] was labelled with digoxigenin (DIG). The information of these ISH probes was listed as below (See Additional file 1). Each mRNA signal was detected from 30 μm parasagittal section with in situ hybridization followed by tyramide signal amplification. Next, we examined the immunohistochemistry for both D1R (1:1000; Frontier Institute, D1R-GP-Af500), Wfs1 (1:1000; Proteintech Group Inc, 11558-1-AP) Neuronal nuclear protein (NeuN) (1:2000; Millipore Sigma, ABN91) in MEC and found the immunoreactivity for D1R match with the signal of Wfs1^+^ cells (Fig. 1E, F). These data indicate that Island cells preferentially express D1R in MEC.

**Fig. 2:**
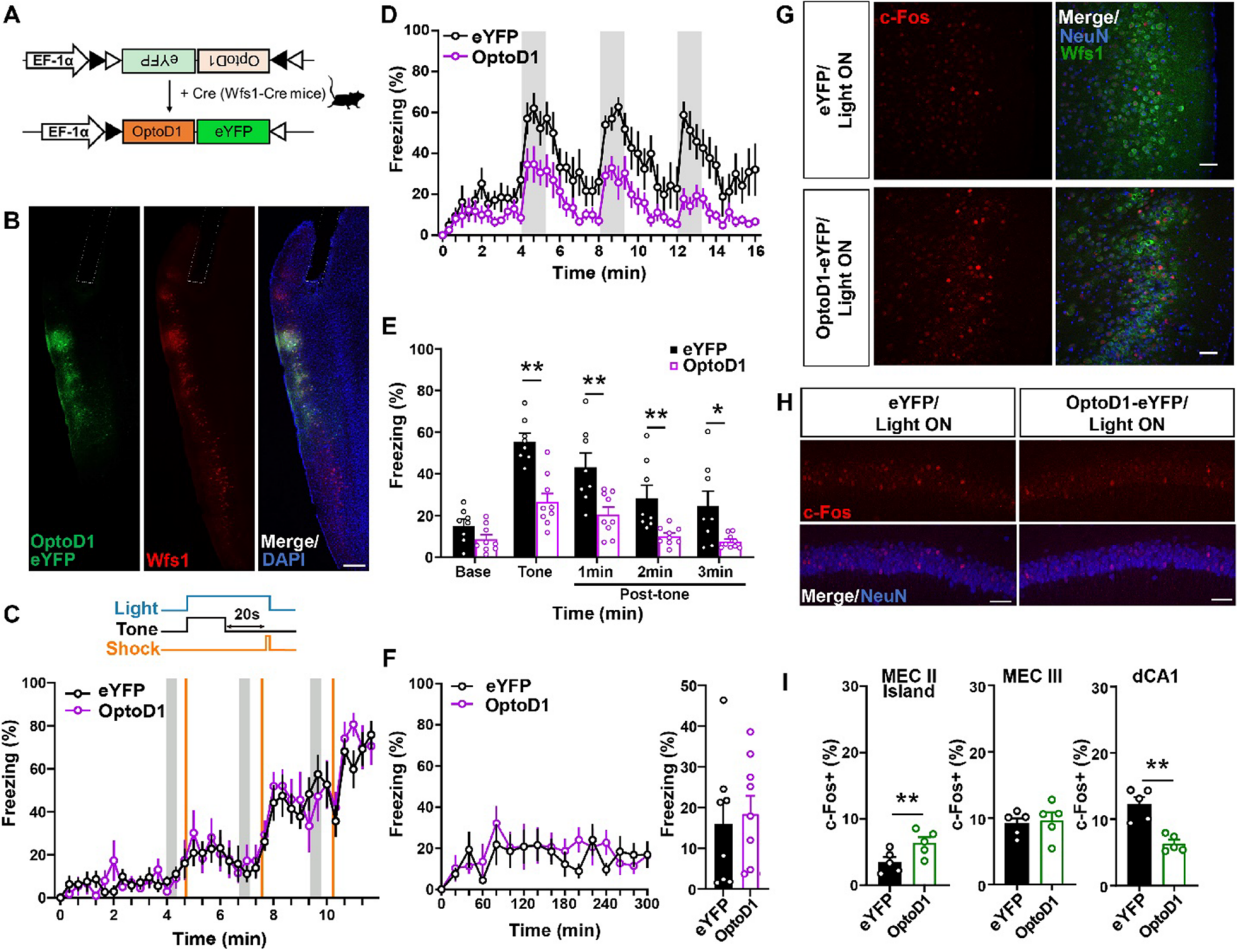
Optogenetic activation of dopamine D1 receptor in Island cells inhibit TFC. **A** Cre-dependent OptoD1-eYFP expression in Island cells of MEC. **B** Parasagittal sections of MEC, which was injected by AAV-EF1a-DIO-OptoD1-eYFP, immunostained with anti-GFP (green) and anti–Wfs1 (red). The white-dotted lines indicate the fiber track. Scale bar, 200 μm. **C** Time course of freezing responses (%) in OptoD1-eYFP (purple) or eYFP (black) groups during the conditioning with a 20-s trace period on day 1. Gray and orange bars represent 20-s tone and shock representations, respectively. Two-way repeated measure ANOVA (n = 9, 8; *F*_(35, 525)_ = 0.6691, *P* = 0.928) **D** Time course of freezing responses (%) during testing session on day 2. Gray bars indicate 60-sec tone representations. Two-way repeated measure ANOVA (*F*_(48, 720)_=1.487, *P* = 0.020). **E** The averaged freezing responses (%) over the three bins during each tone period and that of the first one min, second and third post-tone periods. Unpaired *t*-test, (Tone:* t*_15_ = 5.284, *P* < 0.0001, 1min: *t*_15_ = 3.214, *P* = 0.0058, 2 min: *t*_15_ = 3.218, *P* = 0.0058, 3 min: *t*_15_ = 2.603, *P* = 0.02). **F** Time course of freezing responses (%) during the testing session on day3 in the conditioned context (left). Two-way repeated measure ANOVA (*F*_(16, 111)_ = 0.8329, *P* = 0.6462). The averaged freezing responses (%) (right). Unpaired *t*-test, (*t*_15_ = 0.3620, *P* = 0.7224). **G** and** H** Representative c-Fos staining images in the MEC (**G**) and dCA1 (**H**) of the both groups under the light ON condition. Scale bar, 50 μm. **I** Percentage of c-Fos positive cells in MEC layer II (left), MEC layer III (middle), and dCA1 with Wfs1^+^ (dCA1; right) for OptoD1-eYFP (green) or eYFP (black) groups. (n = 5, 5 mice, total 5124, 10,876 and 2684 NeuN + cells from 15 slices in OptoD1-eYFP; total 4550, 10,429 and 2437 NeuN + cells from 15 slices in eYFP) Unpaired *t*-test, (MECII: *t*_8_ = 2.478, *P* = 0.0038, MECIII: *t*_8_ = 0.3273, *P* = 0.7518, dCA1: *t*_8_ = 4.959, *P* = 0.0011). Mean ± SEM. **P < 0.01, *P < 0.05.

We next examined the roles of the D1R activation on TFC with OptoD1 replacing intracellular domains of rhodopsin with those of D1R for optogenetically activation of D1R–mediated Gs signaling [14]. We bilaterally injected AAV_2/5_-EF1α-DIO-OptoD1-eYFP (UNC Vector Core, AV6284C; 5.4 × 10^12^ gc/ml) [14] or AAV_2/5_-EF1α-DIO-eYFP (UNC Vector Core, AV4310L; 4.0 × 10^12^ gc/ml) as a control into the MEC (AP: − 4.85, ML: ± 3.45, DV: − 3.30) of 10–14 weeks-old Wfs1-Cre transgenic mice [4] and bilaterally implanted the optic fibers (200 μm core diameter, 0.39 NA, Thorlabs) into the MEC (AP: − 4.50, ML: ± 3.50, DV: − 2.00 at 10 degrees anterior-to-posterior) (Fig. 2A, B) as we previously demonstrated [4, 6, 7]. Four–six weeks after the surgery, on Day 1, we subjected them to TFC in which after a 240 s acclimation, a 20 s conditioned stimulus (CS; tone, 5 kHz 80 dB) is followed by a 2 s foot shock as the unconditioned stimulus (US; shock, 1.0 mA, 2 s) with 20 s temporal gap, 3 trials at 3 min intervals as we previously demonstrated (Fig. 2C) [4, 6, 7]. To optogenetically activate the D1R in Island cells of the MEC, we bilaterally delivered the blue light illumination (450 nm, 10 mW) only during the CS-US association periods (total 42 s; 20 s [tone] + 20 s [trace] + 2 s [shock]). On Day 2, we presented the tone three times (60 s, each at 3 min intervals) in different context as a testing session for the memory recall of TFC. On Day 3, we exposed them to the conditioned context for 5 min to examine the memory recall for contextual fear conditioning (CFC). In the testing session on Day 2 for memory recall of TFC, the OptoD1 group showed a significantly lower freezing response during the tone and post-tone period compared with the eYFP control group (Fig. 2D, E). However, there was no difference in the freezing responses between OptoD1 and eYFP groups during the exposure to the conditioned context (Fig. 2F). These suggest that the optogenetic activation of D1R in Island cells selectively regulate TFC.

To demonstrate the effects of the optogenetic activation of D1R in Island cells on the neural activity in the MEC and dorsal hippocampal CA1 (dCA1) during TFC, we perfused them 90 min after TFC and then examined the immunohistochemistry for c-Fos (1:500; Santa Cruz, SC-52, lot: K0515) and NeuN (1:2000; Millipore Sigma, ABN90P) (Fig. 2G, H). We found that c-Fos expression in Island cells of OptoD1 mice was significantly higher than the eYFP control mice (Fig. 2I, left), suggesting that OptoD1 stimulation caused the excitation of Island cells in MECII during TFC. On the other hand, we found that c-Fos expression in dCA1 cells of OptoD1 mice was significantly lower than the eYFP mice (Fig. 2I, right), suggesting that OptoD1 stimulation in Island cells caused the suppression of dCA1 cells during TFC, probably through the feedforward inhibition [4]. We did not observe the effect on c-Fos expression in MECIII neurons (Fig. 2I, middle), suggesting that MECIII cell activity may not depend on the Island cell activity in MEC.

In this study, we found that Island cells preferentially express D1R in the MEC. The optogenetic activation of D1R in Island cells during CS-US association periods during TFC inhibited TFC memory without affecting CFC memory. The optogenetic activation facilitated the c-Fos expression in Island cell while inhibited the dCA1 cells. A previous study shows that dopamine activates the neural activity in the EC, mediated through D1-like and not D2-like dopamine receptors [15]. These results suggest that the activation of D1R in MECII Island cells facilitate Island cell activity, which would inhibit the dCA1 pyramidal cell activity, and then regulate TFC. Further studies will be required to directly investigate the roles of dopaminergic inputs into the MEC to understand the neural circuit mechanism for temporal association learning.

### Supplementary Information

**Additional file 1.** The sequence information on the ISH probes for Wfs1, Reelin and D1R in this fluorescent double ISH experiment, related to Figure. 1A–D.

## Data Availability

Data for analysis will be made available by the corresponding author upon reasonable request.

## Abbreviations

AAV: Adeno-associated virus
CalB: CalbindinD-28 K
D1R: Dopamine D1 receptor
EC: Entorhinal cortex
dCA1: Dorsal hippocampal CA1
GABA: Gamma-aminobutyric acid
HPC: Hippocampus
MEC: Medial entorhinal cortex
NeuN: Neuronal nuclear protein
Wfs1: Wolfram syndrome 1
ISH: In situ Hybridization
FITC: Fluorescein isothiocyanate
DIG: Digoxigenin
TFC: Trace fear conditioning
CFC: Contextual fear conditioning
