# Supplementary material for: Optogenetic activation of dopamine D1 receptors in island cells of medial entorhinal cortex inhibits temporal association learning
Source: Mol Brain. 2023 Nov 14;16:78. doi: 10.1186/s13041-023-01065-3 (PMC10647136; doi:10.1186/s13041-023-01065-3)
Supplement: Supplementary file 1 — Additional file 1. The sequence information on the ISH probes for Wfs1, Reelin and D1R in this fluorescent double ISH experiment, related to Figure. 1A–D. [file 13041_2023_1065_MOESM1_ESM.docx]

**Additional data**

For the detection of D1R, we mixed two antisense probes D1R mRNA probe #1 and #2.

**Wfs1 mRNA probe** (designed by the Allen institute; RP_050303_02_E06):

5′- TACGCCAAGGGCATCATTCCCACCAACCTGTTCCTGCAGGATGAGGATGAAGATGAGGACGAGCTGGCAGGGAAGAGCCCCGAGGACCTGCCACTACGCCAGAAGGTGGTGAAGTACCCTTTACACGCCATCATGGAGATCAAAGAGTACCTGATTGACGTAGCCTCCAAGGCCGGCATGCACTGGCTCTCCACCATTGTACCCACCCATCACATCAACGCCCTCATCTTCTTCTTCATCATCAGCAACCTAACCATCGACTTCTTCGCCTTCTTCATCCCCCTGGTGGTCTTCTATCTGTCCTTTGTGTCCATGGTCATCTGCACGCTCAAGGTGTTCCAGGACAGCAAGGCCTGGGAGAACTTCCGTACTCTCACCGACCTGCTGCTGCGCTTCGAGCCCAACCTAGACGTGGAGCAGGCCGAAGTGAACTTCGGCTGGAACCACCTGGAGCCCTACATCCACTTCCTACTGTCAGTCGTCTTTGTCATCTTCTCCTTCCCGCTGGCCAGCAAGGACTGCATCCCCTGCTCGGAGCTGGCCGTCATCTCCACCTTCTTCACGGCGACCAGCTACATGAGCCTGAGCAGCTCTGCTGAGCCCTATACCAGGCGTGCCCTGGTCACCGAGGTGGCTGCCGGCTTGCTGTCCCTTCTGCCCACCGTGCCTGTGGACTGGCGCTTCCTGAAAGTACTCGGCCAGACTTTCTTCACTGTGCCCGTTGGCCACTTCATCATCCTCAACGTCAGCCTCCCCTGCCTGCTCTATGTCTATCTCTTTTACCTCTTCTTCCGCATGGCCCAGCTGAGGAACTTCAAGGGCACTTATTGCTACCTGGTGCCCTACCTGGTG-3′

**Reelin mRNA probe** (designed by the Allen institute; RP_071018_03_D08):

5′- TCAGCTGGAGAAAATTAGAGCCCCTTCCAATGTGAGCACAGTCATCCACATCCTGTACCTCCCCGAGGAAGCCAAAGGGGAGAGCGTGCAGTTCCAGTGGAAACAGGACAGCCTGCGAGTGGGTGAGGTGTATGAGGCCTGCTGGGCCCTGGATAACATCCTGGTCATCAATTCAGCCCACAGAGAAGTCGTTCTGGAGGACAACCTCGACCCGGTCGACACGGGCAACTGGCTCTTCTTCCCTGGAGCAACGGTCAAGCATAGCTGTCAGTCAGATGGGAACTCCATTTATTTCCATGGAAATGAAGGCAGCGAGTTCAATTTTGCCACCACCCGGGATGTAGATCTTTCTACAGAGGATATTCAAGAGCAGTGGTCAGAAGAATTTGAGAGCCAGCCCACAGGATGGGATATCTTGGGAGCAGTAGTTGGTGCAGACTGTGGAACCGTAGAATCAGGACTATCACTGGTGTTCCTCAAAGATGGAGAGAGGAAGCTTTGCACCCCCTACATGGATACAACTGGTTATGGCAACCTGAGGTTCTACTTCGTTATGGGAGGAATCTGTGACCCTGGAGTCTCTCATGAAAACGATATCATCTTATATGCAAAGATTGAAGGAAGAAAAGAACACATTGCACTGGACACTCTTACCTATTCTTCCTATAAGGTTCCGTCTTTGGTTTCTGTGGTCATCAACCCTGAACTTCAGACACCTGCCACCAAATTTTGTCTCAGGCAAAAGAGCCACCAAGGGTATAATCGGAATGTCTGGGCTGTGGACTTCTTCCATGTGCTGCCCGTTCTCCCTTCAACAATGTCTCACATGATCCAGTTTTCTATTAATTTGGGATGCGGCACACACCAGCCTGGGAACAGCGTCAGCTTGGAGTTTTCTACTAACCATGGACGGTCCTGGTCCCTACTCCACACTGAGTGCTT-3′

**D1R mRNA probe #1** (designed by Sariñana et al. Proc Natl Acad Sci, 2014):

5′-ACAAAAGCACAATGGTGTTCCATCAGGAGCATCTCCATAGCAATCCAAGCCATACCAGGAAGAGAGCCGCTTGCTTTCCACCTGTCTTCTGGGTTCAGTGCTCCAGGTCGCTGTTCCCTGGCATCCGCTGTCCCTAGATTCCCCAAGGAATCATAGGCTTTTAAGCATACTCTAAGAGTCTGGGGCCTCTTCCTGGTCAATCTCAGTCACTTTTGGGGATGCTGCCTCTTCTTCTGAGACACAGCCTAAAATACATGCATTTCTCCTTCAAGCCCCTGGTGCCACATCTCTCCAA ATGCC-3′

**D1R mRNA probe #2** (designed by the Allen institute; RP_050825_01_E12):

5′-CTCATAAGCTTTTACATCCCCGTAGCCATTATGATCGTCACTTACACCAGTATCTACAGGATTGCCCAGAAGCAAATCCGGCGCATCTCAGCTTTGGAGAGGGCAGCAGTCCATGCCAAGAACTGCCAGACCACCACAGGTAATGGAAACCCTGTCGAATGCTCTCAATCAGAAAGTTCCTTTAAGATGTCCTTTAAGAGGGAGACTAAAGTCCTGAAGACACTGTCTGTGATCATGGGGGTATTCGTGTGCTGCTGGCTCCCTTTCTTCATTTCGAACTGTATGGTGCCCTTCTGTGGCTCTGAGGAGACCCAGCCATTCTGCATTGATTCCATCACCTTCGATGTATTTGTGTGGTTTGGCTGGGCGAATTCCTCCCTGAACCCCATTATTTATGCTTTTAATGCCGATTTTCAGAAGGCATTCTCGACCCTCTTAGGATGCTATAGACTCTGCCCTACAACGAATAATGCCATAGAGACTGTAAGCATCAACAACAACGGGGCTGTGATGTTTTCCAGCCACCATGAGCCCCGAGGCTCCATCTCCAAGGACTGTAATCTGGTTTACCTGATCCCTCATGCTGTGGGCTCCTCTGAGGACCTGAAGAGGGAGGAGGCCGGTGGCATACCTAAGCCACTGGAGAAGCTGTCCCCGGCCTTATCGGTCATATTGGACTATGACACCGATGTCT-3′
